# Classification of the Type of Contact Between Primary Molars as an Indicator of the Likelihood of Future Caries

**DOI:** 10.3390/children12020161

**Published:** 2025-01-29

**Authors:** Andrea Cortes, Kim Rud Ekstrand, Sofía Jácome-Liévano, Stefania Martignon

**Affiliations:** 1 UNICA-Caries Research Unit, Research Department, Universidad El Bosque, Bogotá 111321, Colombia; jacomesofia@unbosque.edu.co (S.J.-L.); martignonstefania@unbosque.edu.co (S.M.); 2 Cariology & Endodontics, Department of Odontology, Faculty of Health and Medical Sciences, University of Copenhagen, 2200 København, Denmark; kek@sund.ku.dk

**Keywords:** tooth, deciduous, infant, child, risk factors, dental caries

## Abstract

Background/Objectives: Contact areas between first and second primary molars, shaped by concave/convex proximal surface morphology, are associated with proximal caries with concave surfaces linked to biofilm stagnation. This study aimed to evaluate the accuracy and clinical feasibility of a scoring system for classifying contact types (concave–concave, concave–convex, convex–convex, convex–concave) for dental practitioners. Methods: Following ethical approval and informed consent, 116 4–5-year-old children were enrolled. A calibrated examiner assessed caries on the distal of first molars and mesial of second molars using ICDAS-merged criteria and scored 464 proximal contacts clinically and radiographically. Stone models from silicone impressions were also scored by 17 trained practitioners, who repeated scoring a week later and assessed contacts clinically in six children. Practitioners completed a feasibility and satisfaction questionnaire. Results: Convex–convex contacts were most prevalent (58.6%), followed by convex–concave (32.6%). Contact type significantly correlated with caries (OR = 13.5; 95% CI: 6.4–28.3). Inter- and intra-examiner reproducibility ranged from 0.71 to 0.82. Most practitioners felt very (64.7%) or moderately (35.3%) capable of applying the system, found it low in difficulty (70.6%), and expressed high satisfaction (82.4%). Conclusions: The study indicated that it is possible for dental practitioners to classify the proximal contact types between primary molars both in an accurate and clinically feasible way. The system exhibited high reproducibility and practitioner satisfaction, indicating its potential as a valuable tool for identifying caries-prone surfaces and supporting evidence-based caries management.

## 1. Introduction

Dental caries has been described as a biofilm-mediated, sugar-driven, multifactorial, dynamic disease that results in the phasic demineralization and remineralization of dental hard tissues [1]. This disease remains one of the most prominent chronic diseases worldwide, affecting more than 60% of children. In Colombia, the fourth National Oral Health Survey (NOHS) reported a prevalence of conventional caries experience (dmft > 0) in 5-year-olds of 38%, with the prevalence reaching 40% in the city of Bogotá.

A previous 2-year prospective epidemiologic study demonstrated a high prevalence of clinical caries experience in children aged 4 and 6 years at 59.0% and 66.5%, respectively. When initial stage caries lesions and radiographic findings were included, the prevalence increased to 99.8% and 100%, respectively. The study also identified the most caries-prone surfaces as the distal surface of the primary first molars (20.5% to 35.0% at 4 and 6 years, respectively) and the mesial surface of the second primary molars (14.8% to 21.8% at 4 and 6 years, respectively) [2].

The high caries experience in proximal surfaces can be attributed to the morphological characteristics of primary molar teeth, specifically variations in the contact areas between the first and second primary molars [3] that create surfaces that are challenging to clean with conventional tooth brushing, allowing plaque to accumulate. These complexities in proximal surfaces may play a crucial role in the development of caries lesions in these areas [4]. Carlsen [5], in his textbook, described the approximal surfaces of permanent and primary molar teeth as morphologically convex or concave in both the buccolingual and occluso-cervical directions. Building on this understanding, several systems have been proposed to classify the morphology of approximal surfaces [6,7,8], recognizing that some types of approximal contact areas promote more plaque accumulation than other types, increasing the risk of caries development. Such classifications aim to support preventive strategies for these high-risk surfaces [6,7,8].

The interproximal area (IPA) classification system developed by Cortes and coworkers [6], which utilized occluso-cervical images of resin models, provided a clear and standardized method to assess the concavity or convexity of approximal surfaces. The study demonstrated that the concave–concave morphology of approximal surfaces—specifically on the distal surface of the first primary molar and the mesial surface of the second primary molar—could predict future caries lesions (OR = 15.7 CI 5.1–48.3 *p* < 0.001) compared to a convex–convex morphology (OR = 1). This system shows promise as a diagnostic tool for caries risk assessment. Additionally, the study reported high reproducibility of the morphological classification, suggesting that with adequate training, practitioners can reliably distinguish between concave and convex surfaces.

Building on these insights, caries progression can be categorized using the ICDAS system, which is widely recognized in clinical settings. The ICDAS classification includes three stages, which are Initial (ICDAS 1–2), Moderate (ICDAS 3–4), and Extensive (ICDAS 5–6), which help in the assessment of caries severity based on the visibility of lesions. These stages are crucial for guiding treatment decisions, from preventive care to restorative procedures.

In this study, we sought to build on prior research by hypothesizing that the classification of the IPA between primary molar teeth—previously demonstrated to be a clinical predictor of caries [6]—is both usable and applicable in clinical practice. Despite its promising utility, the IPA classification system [6] has not yet been tested in clinical settings by practitioners. To address this gap, this study aims to evaluate the feasibility and applicability of the IPA morphological classification system, both on stone models and in clinical settings to characterize the interproximal areas between primary molar teeth.

## 2. Materials and Methods

Ethical approval for the study was granted by the Ethics Committee at Universidad El Bosque (UEB PCI-2017–9604). Informed consent was obtained from both the children’s parents and the participating practitioners.

**Figure 1:**
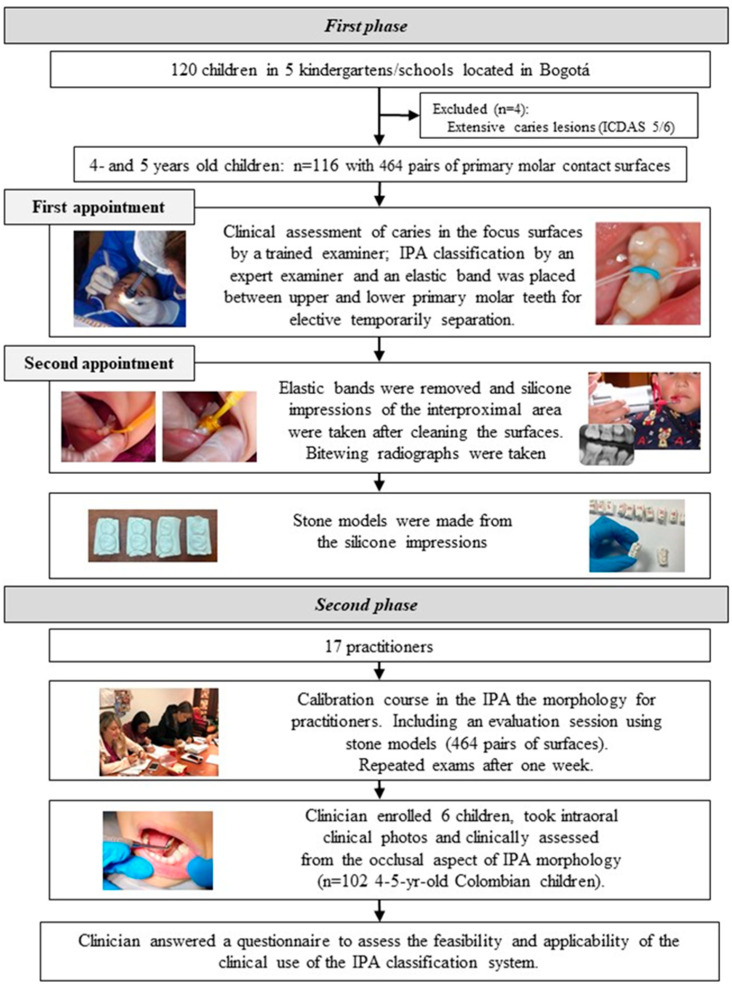
Study flowchart.

The sample size was calculated using the Sample Size Program^®^ (version 1.1), based on the findings of a previous study [6]. The calculation assumed a type I error of 5%, a type II error of 10%, two-tailed tests, and a proportion of 47%. This yielded a minimum required sample size of 96 participants. The study was conducted in two phases (Figure 1).

### 2.1. First Phase

The first phase of the study was conducted in kindergartens and schools located in Usaquén, Bogotá (*n* = 7). These institutions were invited to participate through a visit and a formal letter from the researchers. Five schools agreed to participate, providing space for clinical assessments and facilitating contact with parents. Parents of children aged 4 to 5 years were invited to participate via a letter and were asked to return signed informed consent forms. Out of 120 consent forms received, 116 children were enrolled in the study. Four children were excluded due to extensive cavitated caries lesions (ICDAS score 5 and/or 6) on the distal surface of the first molar or the mesial surface of the second primary molar. These children were referred for caries treatment (Figure 1). The final sample included 116 children, representing 464 pairs of primary molar contact surfaces. Clinical examinations were conducted on-site at each participating school using a portable dental unit.

**Figure 2:**
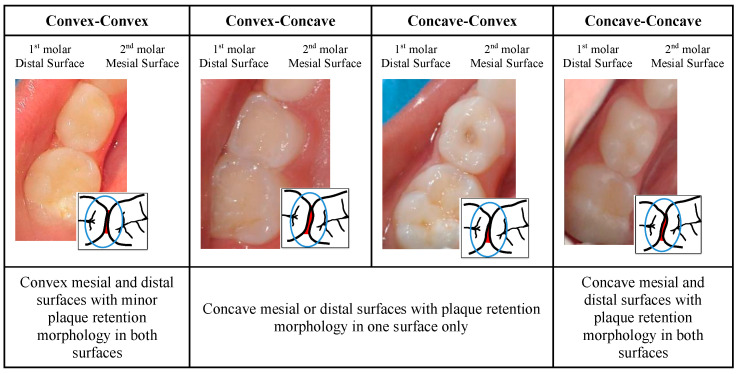
IPA clinical classification from the occlusal aspect of the type of contact between primary molars.

Each child attended two appointments: At the first appointment, supervised tooth-brushing with children’s fluoride toothpaste was conducted, followed by the clinical assessment of caries status on the surface. Afterwards, the type of contact of the mesial surface of the first and distal surfaces of the second upper and lower primary molar teeth on the right or left side was classified from the occlusal point of view by an expert examiner as follows: 0—convex mesial and distal surfaces with minor plaque retention morphology (convex–convex); 1—concave mesial/distal surface with plaque retention morphology on one surface (convex–concave/concave–convex); and 2—concave mesial and distal surfaces with plaque retention morphology on both surfaces (concave–concave), as shown in Figure 2.

At the end of the first appointment, elastic bands were temporarily placed to separate the 1st and 2nd primary molars (upper and lower, right and left) for two days. At the second appointment, held two days later, the elastic bands were removed, and the distal and mesial surfaces of the teeth were thoroughly cleaned. Silicone impressions of the approximal contact areas were then taken, following the procedure described in a previous study [6]. These impressions were cleaned, stored, and subsequently used to create stone models for analysis of the interproximal contact morphology between the first and second molars in the upper and lower jaws. At the end of the second appointment, bitewing radiographs were captured in accordance with the European Academy of Paediatric Dentistry (EAPD) radiographic protection guidelines for children [9]. This was achieved using mobile radiographic equipment (X-PORT II Model, EZX-60^®^ Genoray, Gyeonggi-do, Republic of Korea) and a dental radiography intraoral detector size 1 (Xios Supreme, Dentsply Sirona, Bensheim, Germany) with a bitewing radiograph holder. The radiographs were used to determine the presence or absence of caries and were scored twice by the first author to ensure accuracy and consistency.

### 2.2. Second Phase

Twenty dentists were invited to participate in the study, and 17 practitioners were enrolled. A one-day training course, led by an expert examiner (AC), was conducted to calibrate the practitioners in assessing the contact area morphology between the primary molars of children. The calibration course included an examiner preparation phase and a development phase, which consisted of a theoretical session, pre-clinical practice, pre-clinical examinations, and a discussion of the calibration results. Initially, participants reviewed the previously proposed classification system for approximal surface morphology [6]. The theoretical session featured a PowerPoint^®^ presentation with clinical images illustrating examples of distal surfaces on first primary molars and mesial surfaces on second primary molars. These were classified as convex–convex, convex–concave, concave–convex, or concave–concave. This session allowed for the discussion of specific cases. To ensure that all types of morphology were represented in the pre-clinical sessions, stone models were specifically created for this study. During the pre-clinical practice, practitioners worked with 80 pairs of surfaces from these stone models. In the subsequent pre-clinical examinations, each practitioner assessed 464 pairs of stone models, which had been previously scored by the expert examiner under standardized lighting and spatial conditions. The practitioners repeated these 464 assessments one week later. Examiner reproducibility was defined as achieving a kappa score of at least 0.7; if this threshold was not met, practitioners revisited the assessments after discussing and resolving any doubts. After calibration, each practitioner enrolled six children from their private clinic (a total of 102 Colombian children aged 4–5 years). They took intraoral clinical photos and clinically assessed the occlusal aspects of the contact type. Upon completing these exams, the practitioners filled out a closed-ended questionnaire designed to evaluate the feasibility and applicability of the contact classification system in clinical practice (Table 1). The self-reported questionnaire used a five-point Likert scale.

### 2.3. Statistical Analysis

Unweighted kappa (with 95% confidence intervals) was used to assess the intra-examiner reproducibility of the clinical and radiographic scoring systems. To evaluate reproducibility, the examinations were repeated after one week on 12 randomly selected children, representing 10.3% of the study sample. Unweighted kappa was also applied to determine intra-examiner reproducibility for the morphology classification, using stone models created from clinical impressions. Descriptive statistical analyses were performed to report the caries status based on the clinical and radiographic presence or absence of caries. The caries index included categories for Initial (ICDAS 1–2), Moderate (ICDAS 3–4), and Extensive (ICDAS 5–6) lesions. Variations (mean) were expressed as ±1 standard deviation, and the prevalence of caries was reported as a percentage. Clinical and radiographic scores were subsequently merged, with the most severe score used to represent the severity or depth of the caries lesion, as described in previous studies [2]. The relationship between the different types of contact and the presence of caries (as the dependent variable) was analyzed using odds ratios, with corresponding 95% confidence intervals. Statistical significance was set at a threshold of 5%. For the 17 dentists participating in the study, inter- and intra-examiner reproducibility was assessed during the calibration course based on the first examination. To evaluate inter-examiner reproducibility throughout the study, an expert examiner assessed the contact morphology type using photographs. Descriptive statistics were employed to analyze the feasibility and applicability of the type of contact classification system in clinical practice, as reported by the practitioners.

## 3. Results

### 3.1. Reproducibility for the Authors

The weighted kappa values for intra-examiner reproducibility were 0.76 for clinical caries assessments, 0.84 for radiographic caries assessments, and 0.81 for the type of contact assessment.

### 3.2. Association Between the Clinical and Radiographical Caries Status and the Type of Contact Between Primary Molars

Out of the 464 surface pairs evaluated, 278 (59.9%) had no caries lesions and 133 (28.6%) exhibited initial caries lesions on one surface. Table 1 describes the relationship between adjacent tooth surface morphologies and the prevalence of caries lesions on one surface. Concave–concave surfaces show the highest risk of caries, with an odds ratio of 13.5 (95% CI: 6.4–28.3).

### 3.3. Reproducibility for Practitioners on Stone Models

The practitioners’ intra-examiner reproducibility weighted kappa values were between 0.72 and 0.81, and corresponding values for the inter-examiner reproducibility were between 0.71 and 0.75.

### 3.4. Practitioners’ Feasibility and Applicability of the Classification of Primary Molars—Type of Contact

Each of the 17 trained practitioners classified the type of contact morphology in six children, resulting in a total of 102 children enrolled and 24 assessments per practitioner. Intraoral photos were taken by the dentists, yielding 408 photos for evaluation by the expert examiner. The examiner’s intra-reproducibility, assessed by re-examining 25% of the photos, had a weighted kappa value of 0.81. The agreement between each practitioner and the expert examiner was calculated, with a mean kappa value of 0.76 ± 0.03.

Regarding the clinical application of the type of contact classification, practitioners reported feeling very capable (64.7%) or moderately capable (35.3%). Most practitioners found the process to involve little difficulty (70.6%) or some difficulty (23.5%). Furthermore, 82.4% of practitioners felt completely satisfied, 11.8% felt slightly satisfied, and all expressed complete satisfaction with using the classification system (Table 2).

## 4. Discussion

This study implemented and trained seventeen practitioners for the use of the IPA classification of primary molars by the type of contact (interproximal area/type of contact) [6] and evaluated the feasibility and applicability of the classification system based on the experience of the practitioners. Implementing this classification during clinical assessments offers another indicator that can predict future caries lesions, supporting specific homecare and in-office preventive approaches [6].

This is the first study in which the type of contact classification between primary molars was measured clinically by dentists. A previous study [6] reported the classification of the type of contact of adjacent primary molar teeth using stereomicroscopic images of models taken from an occlusal–cervical direction, which was based on the concept that concave interproximal surfaces are more likely than convex surfaces to accumulate plaque. The study also reported the highest risk for developing caries on the concave–concave surface (OR = 15.7 CI = 5.1–48.3 *p* < 0.001), and the results in this study showed similar figures (OR = 13.5 CI = 8.25–21.27 *p* < 0.0001).

Kirthiga et al. [10] introduced the OXIS classification, a three-dimensional system for interproximal contact areas between primary molars, which includes Open (O), X-shaped (X), I-shaped (I), and S-shaped (S) contacts. A related study [7] assessed OXIS contacts in three- to four-year-old children, finding that most children exhibited more than one type of contact in different quadrants, with the I-shaped contact being the most prevalent (75.5%). Similarly, a retrospective study using CBCT images of 3- to 10-year-old children reported OXIS prevalences of I (79.7%) and X (10.0%), with an almost perfect correlation (kappa = 0.958) between CBCT images and clinical photographs [10].

In addition to the IPA classification system [6], the OXIS system demonstrated that type S is the most susceptible to proximal caries due to its complex morphology [11]. Furthermore, the shapes of the marginal ridges in contacts between primary molars with concave morphologies are significantly associated with caries lesions that extend into the dentin [12]. The training and calibration of examiners ensure accurate diagnoses from the early stages, enabling the timely implementation of preventive treatments. Previous studies have demonstrated that calibration processes significantly benefit health professionals by enhancing diagnostic consistency [13], ultimately improving outcomes for patients. This study conducted several reliability assessments using categorical data, for which Cohen’s kappa is the recommended method for expressing the level of agreement [13]. Since the kappa values were greater than 0.61, it can be concluded that there was at least substantial agreement in all assessments.

For this study, the authors developed a questionnaire to assess the feasibility and applicability of using the type of contact classification system in clinical practice, following current best practices in caries care for children [14]. Although two external researchers in the field of cariology were asked to independently assess each item for coherence, syntax, and semantics, offering suggestions for improvement, no formal validation process was conducted for the questionnaire [15,16]. Nonetheless, the 17 trained practitioners reported feeling capable and satisfied with using the classification system in clinical practice, and they emphasized the importance of assessing the type of contact classification in their patients.

The findings from this study suggest that the type of contact classification should be incorporated into individual caries risk assessments for children. The interproximal contact area between the first and second primary molars plays a role in determining whether these surfaces are prone to caries. Therefore, any classification of the approximal surfaces should be utilized during clinical assessments to help practitioners predict future caries lesions. Evaluating this risk factor contributes to interventions aligned with best-practice caries management guidelines, such as ICCMS™ [14] and, more recently, the CariesCare International model [17]. These guidelines advocate a patient-centered risk-based approach to caries management that preserves tooth structure and maintains oral health in the long term, in line with the current understanding of caries. For an effective assessment, it is necessary to teach children and parents proper hygiene practices, monitor their habits, and control and measure the outcomes through a valid interval; parental involvement also plays a key role in reinforcing oral hygiene habits at home.

However, there are some limitations to this study. First, stone models were used in the type of contact morphology training course, which although based on child models to simulate clinical reality, did not allow for a direct clinical examination of the type of contact. Ideally, practitioners would examine these contacts clinically, but the study was constrained by the fact that only four surface pairs could be assessed per child, requiring a larger number of children and practitioners. Additionally, while practitioners performed the clinical morphological assessments, the expert examiner evaluated the results on a computer screen. Despite practitioners’ efforts to position the surfaces correctly under the camera from the occlusal view, the assessment was based on a single two-dimensional angulation, which limited the ability to adjust the perspective. The future of dental diagnostics could be significantly enhanced by the use of advanced scanning technologies. Devices such as intraoral scanners [18], 3D optical scanners, and laser scanners offer numerous advantages [19], including high-resolution, non-invasive, and quick scans that provide precise reproducible 3D models of dental surfaces. These models can be easily stored, shared, and monitored over time, improving the assessment of caries risk and the effectiveness of preventive measures. Additionally, digital scanning reduces the need for physical models, cutting down on time, cost, and material use. As technology advances, these scanning devices are expected to become more accurate, affordable, and integrated into clinical practices, ultimately improving early detection and patient care.

## 5. Conclusions

This study highlights the feasibility and clinical applicability of using a contact classification system as an effective tool for assessing caries risk in children. By evaluating the interproximal contact areas between primary molars, practitioners can more accurately predict the likelihood of future caries lesions and design targeted preventive interventions. While the findings support integrating this classification system into individual caries risk assessments, further research utilizing advanced 3D imaging and direct clinical evaluations is necessary to improve its accuracy and broader applicability. Incorporating this classification system into caries management guidelines has the potential to enhance patient-centered care, promote more effective prevention strategies, and support long-term oral health preservation.

## Figures and Tables

**Table 1:** Analysis of caries in the first and second primary molar teeth in relation to interproximal morphology with odds ratio (OR), 95% confidence interval (CI), and *p* values.

Adjacent Surface	*n*	Caries Lesions on One Surface	OR	CI	*p* Value
Convex–convex	272	33 (12%)	1	-	-
Convex–concave/ concave–convex	152	74 (49%)	6.87	4.2–11.1	<0.0001
Concave–concave	40	26 (65%)	13.5	6.4–28.3	<0.0001

**Table 2:** Feasibility and applicability of practitioners regarding the clinical classification of the type of contact between primary molars.

Question		Answer	*n*	%
Feasibility	How confident did you feel about your ability to assess clinically the types of contact between molars, from the occlusal point of view, in your patients?	Very capable	11	64.7
		Moderately capable	6	35.3
		Neither capable or incapable	0	0
		Moderately incapable	0	0
		Very incapable	0	0
Applicability	How satisfied did you feel using the clinical type of contact between primary molar classification in your patients?	Completely satisfied	17	100
		Slightly satisfied	0	0
		Neither satisfied or dissatisfied	0	0
		Moderately dissatisfied	0	0
		Very unsatisfied	0	0
Feasibility	How much difficulty did you have to assess clinically the type of contact between primary molar classification in your patients?	No difficulty	4	23.5
		Little difficulty	12	70.6
		Some difficulty	0	0
		Quite difficult	1	5.9
		Much difficulty	0	0
Feasibility	How satisfied were you with the time it took to assess clinically the type of contact between primary molar classification in your patients?	Completely satisfied	14	82.4
		Slightly satisfied	2	11.8
		Neither satisfied or dissatisfied	1	5.9
		Moderately dissatisfied	0	0
		Very unsatisfied	0	0
Applicability	How important do you consider assessing clinically the type of contact between primary molar classification in your patients?	Very important	16	94.1
		Moderately important	0	0
		Neither important nor not important	1	5.9
		Less important	0	0
		Very unimportant	0	0

## Data Availability

The data generated and analyzed during this study are not publicly available but are available from the corresponding author upon reasonable request and with permission from Universidad El Bosque. Requests for data access will be reviewed to ensure compliance with applicable data protection and ethical regulations. The participants’ information was informed to each participant. The results of this study will be communicated by publications and presentations in international conferences. Only the authors will have access to the final trial dataset.
